# The Medical Association of Georgia

**Published:** 1882-04

**Authors:** 


### Editorial.

### THE MEDICAL ASSOCIATION OF GEORGIA.

The Medical Association of Georgia will hold its thirty-third annual session in this city, beginning on the nineteenth instant and continuing three days.

The near approach of this event affords a fitting opportunity for offering a few observations, concerning the importance of this organization to the medical interests of the State and the obligation which rests upon the members of the profession to yield it a cordial and liberal support.

It will scarcely be denied that some sort of State organization is essential; even to a disinterested and casual observer it must be obvious that combination is necessary to mutual protection and to real progress. Questions of moment frequently arise in connection with all vocations, which require for authoritative settlement the united voice of the representatives of the calling. Assuming then, that upon this point there is no dissension, the practical question is, does this Association fulfill its mission? Does it accomplish all that it ought to, or that it can accomplish?

While, perhaps, it does the best that it can under the attendant circumstances, we must admit that its possibilities seem to us to be great and that its achievements do not appear to be commensurate with its opportunities.

Space forbids a thorough review of the history of the Association, and it would weary the reader for us to recount its toils, its trials and its triumphs, or to follow it in its successes and reverses through a checkered career, covering now the third of a century. Nor is this tedious retrospection necessary for the purposes of this article. The pressing question which demands our attention is the present status of the body.

In our opinion many circumstances, apparently unimportant in themselves, combine to cripple and to impair the usefulness of the Association; and, without citing these causes in detail, we will venture to indicate the points in the policy of the society which, according to our belief, require and ought to receive the most zealous attention.

In the first place, the finances should be placed upon a firm basis, or, in other words, to borrow a metaphor form our political friends, the "financial plank" should be strong. Without substantial support no enterprise of this character can succeed. It is fundamental and indispensable. The treasury had better be over-burdened than inadequately supplied.

In the second place, the law of the organization, whatever it may be, should be impartially, equitably and rigidly enforced. There is no more important office in the gift of this body, than that of censor. The duties may be, and sometimes doubtless are extremely disagreeable, involving labor and sacrifice, but the welfare of the Association largely depends upon their conscientious and intelligent performance—upon the faithful execution of the law. Then if offense is taken it will be because of a duty done, not on account of duty neglected. And, thirdly, the proceedings should be made so interesting and instructive as to invite a large attendance and to secure, thereby, an annual augmentation of the membership.

The last suggestion is, possibly, the most difficult to put into successful operation, as it requires the constant concurrence of a large number of persons, but the propositions submitted are mutually helpful, and the strict observance of any one of them will render the others easier of accomplishment, and pave the way for subsequent reforms.

Another point is worthy of the most serious attention; that is, whether our form of organization is promotive of the best results, and whether it is possible under our present organic chart to be more than a semi-scientific and loosely organized body. It is very doubtful if improvement in this direction is within reach, but we invite attention to the matter in the hope that those most nearly interested will study the subject thoroughly in all its bearings, and be the better prepared to propose such remedies, when the right time comes, as the exigencies of the case shall appear to demand.

So far as the duty of the profession to the Association is concerned, we do not hesitate to express the conviction that it is incumbent upon every respectable practitioner within the State to align himself with the State organization, and to exert his talents and his influence in its behalf.

As a member of the profession, his allegience is due to the representative organization of that profession, and it should be declared, if not by an active, at least by a nominal adherence.

In conclusion, we will add that a large and enthusiastic meeting, animated by a sincere desire to advance the interests of the Association, will afford the best opportunity to put into practical operation any reform that may be determined upon. Let us have such an assemblage on the nineteenth. We, expectant, await with pleasurable anticipation the occasion.

